# Clarifying the Role of Schools in Tendency or Lack of Tendency Toward Smoking among Teenage Boys (11-14) in Mashhad, Iran

**DOI:** 10.5812/ircmj.12848

**Published:** 2014-01-05

**Authors:** Hamid Reza Mohaddes Hakkak, Mohammad Hossein Taghdisi, Davoud Shojaezadeh, Saharnaz Nedjat, Nooshin Peyman, Ali Taghipour

**Affiliations:** 1 Department of Health Education and Promotion, School of Public Health, Tehran University of Medical Sciences, Tehran, IR Iran; 2 Department of Epidemiology and Biostatistics, School of Public Health, Knowledge Utilization Research Center, Tehran University of Medical Sciences, Tehran, IR Iran; 3 Department of Health and Management, School of Health, Mashhad University of Medical Sciences, Mashhad, IR Iran; 4 Department of Biostatistics and Epidemiology, School of Health Mashhad University of Medical Sciences, Mashhad, IR Iran

**Keywords:** Smoking, Adolescent, Tobacco, Schools

## Abstract

**Background:**

Recent studies show that the prevalence of tobacco use among teens and students is increasing, and the initiation age of tobacco use has decreased.

**Objectives:**

The current research aimed to signify the role of schools in the process in which student teenage boys became smokers in 2012 in Mashhad.

**Materials and Methods:**

The current study was part of a qualitative research conducted by content analysis method and purposive sampling, performing 35 in-depth interviews, and 2 focused group discussions. The participants in this research included teenagers, teachers, students` parents , psychologists, and experts in the field of fighting against tobacco use, those who either had the experience of exposure to cigarettes at school, or were well-informed persons about tobacco use.After performing each interview, the interview was transcribed, and analyzed before the next interview. The data were under continuous consideration and comparative analysis in order to achieve data saturation.

**Results:**

After analysis and codification of data, four concept categories were achieved to clarify the role of schools in student smoking: 1) School purity or impurity to high-risk behaviors; 2) Directive or nondirective schools for controlling tobacco; 3) Preventive or predisposing schools for smoking behavior, and 4) Perceived positive outcomes from smoking at school. Each main category was divided into three subordinate themes.

**Conclusions:**

With regard to decrease of cigarette use initiation age and the great influence of schools on teenagers’ behavior, it is recommended to perform special screening programs based on the achieved themes in this research to reduce tobacco use. It is also suggested that school staff pay more attention to students’ communication networks and pressures that are imposed on a student for smoking cigarettes during the school time.

## 1. Background

Smoking is the most important cause of preventable death in the world (1, 2). Every year, six million people die because of the adverse effects of tobacco use (3, 4). According to the World Health Organization reports, in the recent years one person has died every six seconds because of these effects (5). It is estimated that in 2020, use of cigarettes will cause the death of more than 7.5 million people (4); and by 2030, the number will be more than 8 million adults worldwide (6). As half of these deaths happen in middle-aged people, it can lead to a reduced life expectancy of up to 20 - 25 years for 35-61 year old persons (7, 8). The studies show that Tobacco use is considered as one of the important risk factors that increase the general burden of diseases in the world, especially regarding chronic and non-communicable diseases such as cardio-vascular respiratory diseases, cancer, and brain failure (9, 10). The collected data from different studies conducted in Iran show that about 12% of adults at the age 15-99 are smokers (Male 25%, and Female 1.4%) (11, 12).

Considering the Global Youth Tobacco Survey (GYTS), findings in the current decade indicate the decrease of smoking initiation age, and the increase of cigarette use prevalence among teens (13). According to GYTS results, in Iran, 17.5% of students (23.7% boys and 11% girls) had experienced smoking (11). In a study by Kelishadi et al. on 11-18 year- old students in 20 areas of Iran, 14.3% of students were cigarette smokers (14). According to the above-mentioned results, the necessity to consider tobacco use topic among teenagers seems essential, more than ever.

Many experts have considered cigarette use as an introduction to tendency to use other substances, and mention it as the gate to narcotics use (15). With regard to the problems and adverse effects of cigarettes on health, economic, and social fields (6), the necessity to design and execute programs of first level prevention in order to control cigarette and tobacco dependency seems essential. Therefore, with regard to the increase in prevalence of cigarette use among students, and effectiveness of school environments on this matter, we decided to employ a qualitative study to clarify the role of schools and determine the main concepts related to schools when discussing tendency among teenagers. Since women smoking is considered as a stigma in the Iranian culture (12), and with regard to the higher prevalence of cigarette use among Males (23.4%) compared to Females (1.4%) (12), studying the cigarette use among boys became the priority of this research. Therefore, the current study as part of a more comprehensive study with the aim of clarifying the influential role of schools in cigarette smoking initiation was carried out among boy guidance school students in Mashhad city, Iran in 2012.

## 2. Objectives

The current research aimed to signify the role of schools in the process in which student teenage boys became smokers in 2012 in Mashhad.

## 3. Materials and Methods

The current study was part of a qualitative research conducted by content analysis method, and purposive sampling in 2012. The content analysis is beyond extraction of visible content taken from textual data. By this method, in the current study we could obtain contents and hidden patterns from the participant’s data (16).All the researchers in this study were faculty members of universities of medical sciences in Iran and were PhD holders.

### 3.1. Participants

The participants in this research included teenagers, teachers, students’ parents, psychologists, and experts in the field of fighting against tobacco use. Participants were selected based on their experiences and the research objectives. Inclusion Criteria of this study were age 10-35 years, and either cigarette use background in teenage, or having experience about teenagers smoking. For a better understanding of smoking behavior, both teenagers (10-19 years) and adults (20-35 years) were interviewed. Teenagers were two groups including smoking teenagers and non-smoking teenagers. The non-smoking teenagers were selected because by comparison of their conditions and reactions in similar situations, we could achieve a better understanding of the process in which these teenagers became smokers.

Adults included persons who had started to smoke from their teenage years (10-19 years). Adult smokers were selected because by keeping aloof from teenage experience, they possessed a deeper understanding of their experience, and could provide in-depth information in comparison with teenagers, about how they became smokers, and their information was complementary to the information received from the teenagers. In order to create maximum variety in the samples and perceive broader dimensions of this subject, the experts of the field and the students’ parents were involved in this research. Lack of tendency toward continuing the cooperation, presenting dishonest answers and revoking conscious satisfaction, were considered as the exclusion criteria.

### 3.2. Sampling Method

Purposive sampling through Maximum variation sampling was initially used for data collection. In this sampling method, the basis of selecting participants was having special information about the considered phenomenon, and the aim of their selection was to collect these data. In this section, the persons who had the experience regarding smoking behavior and were close to this group in terms of age, or had experience and skill in the field of working with teenagers participated in this research. Samples were selected from different places like Parks, extracurricular classes of the municipality, training associations, schools, universities, drug stop clinics, and Nicotine Anonymous (NicA). Because of the authors` background in working with centers of drug abuse treatment and smoking cessation clinics, the first samples were selected from these centers, and other samples were selected using snowball method. Some of these samples were also selected using accessible samples and direct observation of teenager smoking by the researcher.

### 3.3. Data Collection

The data in this section was collected using in-depth interviews and Focus Group Discussion (FGD). All of the interviews were recorded and transcribed. The interviews were conducted by the first author who was identical with the studied group in the light of gender and regarding the age was also closer to them. He had also activity background in the field of tobacco studies and studies about students and teenagers as well. He had good communication skills and specialty about interview. Interviewees’ written consent was obtained prior to recording their voices, and the research aims were explained to them. In addition, they were assured that all of their information will remain confidential, and only the researcher will have access to their information and sound file. All of the participants studied and signed the form of conscious satisfaction designed by the research team. This conscious satisfaction form had been prepared on the basis of ethical codes regarding work with human cases and approved by Ethics Committee of Tehran University of Medical Sciences (registered under 17878-73530Dated27/2/91).

The places of interview were determined based on agreement of both parties (parks, schools, offices, Therapeutic Community Centers, Methadone Therapy Clinics). The interviews were conducted in face-to-face approach and in an open and semi-structuralized method. The interviews were carried out just with the presence of researcher and the interviewee and not anyone else. The interviews commenced by questions regarding age, education, and leading questions to enter discussion. The key question for smokers was their story about smoking the first cigarette, which they were asked to talk about. The key questions were: “Let us talk about the story of your first smoke. Why did you choose to smoke for the first time? Why made you continue smoking?”

The other questions were related to cases that the interviewee did not mention school conditions, smoking at school, and so on. Moreover, in the new and supplementary interviews, the information of the previous interviews was used, and the new questions were added to the new interview. The new questions were added based on the obtained memos. In addition, the comments made by smoking teenager, parents, experts of teenage behavioral training, teachers, and health trainers of schools were obtained through group discussion .The time of interviews was different ranging from 20 minutes up to 90 minutes. Totally 40 samples were selected for interview that five persons were left out according to lack of interest to continue cooperation , and by interviewers’ discretion. In the focus groups, nobody was left out of this study.

In the 35th interview, we reached data saturation. The researchers did not feel any necessity to repeat any of the interviews. Thirty-five people participated in the interviews, and 15 people took part in two group discussions. The time of each group discussion session was 180 minutes.

### 3.4. Data Analysis

The analysis was carried out by means of Graneheim & Lundman approach (17) (Table 1). 

**Table 1.** The Summery of Steps of Analysis Based on Graneheim and Lundman’s Approach

Steps	Activity
**Start Point**	The interviews were transcribed
**Step1**	The texts were read through several times to get a sense of the whole
**Step2**	Meaning units were extracted from the text.
**Step3**	An abstraction of the meaning units into codes was created.
**Step4**	The various codes were read and re-read and compared against each other. Based on this reading and a reflective process the codes were sorted into sub-categories.
**Step5**	The next step in the analysis was to count the occurrence of each sub-category in the interviews.
**Step6**	The sub-categories were compared with each other and with the original text to create mutually exclusive categories.
**Step7**	An independent analysis of all the texts was performed by each of the two co-authors. All authors discussed the categorization and the content of the categories and consensus about the categorization was reached.

Data collection and analysis were done simultaneously (18). After each interview, the interview tape was transcribed and analyzed prior to the next interview. For data analysis, at first, the interview text was studied several times, and then, important sentences were highlighted and codified.Data were considered ,and comparative analysis was performed in order to extract primary codes. Primary codes could include abstraction of the content (17). In the next stage, themes were organized based on their concept in separate categories. In this part, the primary codes were classified based on differences and similarities (17) in abstract categories and key concepts (19). Continuous and comparative analysis continued until data saturation. The continuous analysis of data began from the beginning of codification, and continued until the end of data collection. Four of the authors participated in data coding process. The maxqda10 software was employed for transcription, classification, and analysis of codes.

During this study, to make sure of credibility, dependability, fittingness, and conformability methods were used for exactitude in qualitative scientific researches (20). The written copies, were codified, studied again, and compared with initial codification results a few days after codifying. In some cases, sample codes were given to interviewees in order to obtain their opinion about harmony of the codes with their statements. In addition, some sections of the transcriptions together with extracted codes were sent to a number of experts to consider the analysis process and determine its accuracy. Finally, themes and categories of different interviews were combined and a description of the quality and effect of the school related causes on the smoking behavior in teenagers was obtained.

## 4. Results

The analyses were carried out in two groups, the Encounter Group, and the Informed persons Group. The encounter group included teenagers either smoking, exposed to cigarette offers, or had experiences about their friends who had became smokers, and also adults who had started smoking from their teenage and education period. The total number of these persons was 35 people (Table 2). 

**Table 2.** The Demographic Data of Studied Patients (Encounter Group)

Characteristics	Values
**Sex, No. (%**)	
Male	35 (100)
Female	0 (0)
**Smoking, No. (%)**	
Yes	23 (65.7)
No	12 (34.3)
**Educational Level, No. (%)**	
Primary School	17 (48.6)
Guidance School	13 (37.1)
High School	5 (14.3)
**Age, y**	
Lowest	11
Highest	32
Mean	16
**Age of Initiation of Smoking, y**	
Lowest	10
Highest	15
Mean	12.4

The informed persons group included four persons from teenagers’ parents, three persons were teachers active in training matters, three persons were health trainers of schools, two persons were psychologists in the field of drug abuse, and three persons were experts of health education and health promotion who worked on the field of tobacco use. The number of these persons was 15.

After analysis and codification, the manner of school effect on students’ smoking behavior was determined as follows:

•Purity or impurity of school in terms of high-risk behaviors;

•Directive or nondirective schools for controlling tobacco; 

•Preventive or predisposing schools for smoking behavior, and

•Perceived positive outcomes from smoking at school (Table 3) 

Then, each main category and subordinate themes of each category were explained.

**Table3.** Main Categories and Subordinate Themes Obtained by Data Analysis

Main Categories
**Impurity of school in terms of high-risk behaviors**
Presence of smoking students
Easy access to cigarettes
Presence of students with high risk behaviors
**Nondirective school for controlling tobacco**
Lack of enforcement or ineffective enforcement of anti-tobacco educational programs
Lack of cooperation of teachers and head masters in executing programs
Lack of families’ participation in executing the programs
**Predisposing school for smoking behavior**
Out of sight places and without supervision inside the school
lack of protective programs for weak students
Smoking teachers
**Perceived positive outcomes from smoking at school**
Cigarette use as a value among students
Obtaining permission of entry to forbidden informed groups at school
Smoking for achieving higher marks

### 4.1. Purity or Impurity of School to High-Risk Behaviors

We considered the pure school as a school in which the students were not exposed to high-risk behaviors. This category had three themes explained as follows.

#### 4.1.1. Presence of Smoking Students

One of the causes emphasized by participants in this study was the influence of school environment on increasing the smoking possibility of teenagers. The presence of smoking students was one of the cases that the participants mentioned as a persuasive factor for cigarette consumption. Participant number 14 in this case said: “Before the beginning of the class or at breaks, the guys who had experienced cigarette consumption described it to us in detail, in a way that you wished to experience it right away” (Male, 19 years old).All of the participants in this research confirmed that the presence of smoking students at school creates more pressure on teenagers for smoking. With regard to the effect of peers on teenagers in this period of life, the pressure will be applied through temptation, and offer in the presence of other students by smoking students.

#### 4.1.2. Easy Access to Cigarettes

Most of the participants expressed that easy access to cigarettes at school, and sales of cigarettes around the school increase the students` possibility of smoking. Some of the interviewees mentioned the presence of smoking students at school, and possessing several cigarettes as one of the factors that leads to easy access to cigarettes. Participant number 17 said: “I smoked my first cigarette at school. One day, we did not have a teacher, and gathered with some guys behind the playground. One of the guys was talking about his smoking. I said: if there is a cigarette right now, I will smoke, too. I hoped that there would not be any cigarettes. He went into the class and brought the cigarette and matches that were inside his bag. It was disgraceful if I did not smoke” (Male, 17 years old).

Generally the participants` comments emphasized the point that easy access to cigarettes at school, could complete the chain of pressure, temptation, and offer, that consequently places the students in an irreversible situation for smoking their first cigarette.

#### 4.1.3. Presence of Students With High Risk Behaviors

Presence of students with other high-risk behaviors like drug abuse or drinking alcohol also creates a collection of high-risk actions including smoking among other students. One of the interviewees who was currently giving up his addiction told us his story:

“Once, one of the guys said: “My father drank wine last night, a little wine has remained at the bottom of his bottle.” He had brought it to school, and we drank it together. During the break, we went behind the school trees, drank the wine, and then smoked a cigarette” (Mail, 21 years old, number 6).

Comments of experts and students’ parents confirm the matter that the presence of students with high-risk behaviors can increase the possibility of smoking among other teenagers. From the view point of the experts who participated in this research, presence of students with high-risk behaviors like drug abuse or drinking alcohol has an effective role on increasing the possibility of the other students’ smoking. This can take place through different ways such as normalization, influence of the high-risk group, and coherence of high-risk behaviors.

### 4.2. Directive or Nondirective School for Controlling Tobacco

Research participants have considered a school as “Directive” when the school staff and the students’ parents effectively participated in the enforcement of anti-tobacco programs. “Nondirective school” consisted of three subordinate themes as follows:

#### 4.2.1. Lack of Enforcement or Ineffective Enforcement of Anti-tobacco Educational Programs

Presence of compiled and purposive educational programs at schools to prevent and control smoking behavior are some of the factors that greatly affect tobacco use control, the teenagers and the experts who participated in this study believed that lack of the enforcement of these programs can make the students vulnerable to smoking behavior. The participants expressed that in Nondirective schools, anti-tobacco educational programs are not priorities. One of the school health trainers explained the subject in this way:

“The authorities of these schools do not consider any priority for anti-tobacco programs.” (Male, 36 years old)

Ineffective enforcement of available programs is also another reason mentioned by experts in this regard. Participant number 45, an expert of health education, expressed:

“The selected educational methods are not proportionate to educational aims.”(Male, 30 years old, health educator)

Irrelevancy of educational methods with educational goals, and the low quality of education are the cases that all experts mentioned as a failure cause of anti-tobacco instructional programs at schools. It seems that lack of benefiting from experts and educational planners in designing and executing instructional programs has great influence on the failure of such programs.

#### 4.2.2. Lack of Cooperation between Teachers and Head Masters in Executing Programs

According to the participants` statements, another considerable subject is the teachers` important role in prevention of tendency toward cigarettes. 

Participant number 30 mentioned: “None of the teachers preached or said what to do in cases of cigarette offerings” (Male, 14 years old).

Lack of appropriate participation of head masters and school authorities in such programs is another problem that makes a school “nondirective”. In addition, a health trainer states, “You cannot do anything at school if the head master does not want you to”. In this case, everything will fail” (Female, 37 years old, school health trainer).

#### 4.2.3. Lack of Families’ Participation in Executing the Programs

The participants confirmed that if the students’ parents do not cooperate with schools appropriately, playing a directive role by schools is impossible. One of the teachers said in this regard:

“Many families do not agree to send their teenagers to schools for extracurricular non-educational training workshops” (Female, 35 years old, teacher).

Lack of cooperation and participation of families in anti-tobacco instructional programs was the concern of all the experts participating in this research. According to these comments, enforcement of anti-tobacco instructional programs will face failure or achieve low output, without appropriate cooperation of the students’ families.”

### 4.3. Preventive School or Predisposing School for Smoking Behavior

Preventive schools are those that do not create grounds for cigarette use among students and eliminate or control the conditions that reinforce the behavior, and tend to remove the use opportunities and temptations, or minimize them.

This category has three subordinate themes as follows:

#### 4.3.1. Out of Sight Unsupervised Places Inside the School

Interview with persons that have had a chance to smoke at school, shows that, schools can provide chances for teenagers to smoke. Availability of the out of sight unsupervised places inside the school, or undefined class hours without teachers are some of the opportunities provided for students to engage in high risk actions. Participant number 16 said:

“Behind the precinct of our school, there was a back yard that teachers parked their cars. One day I went there and saw that my friends were smoking. They asked, “Do you smoke?” I replied, “Yes, I do” (Male, 16 years old).

With regard to the fact that the influence of peer groups on teenagers is very high, being exposed to the situation increases the possibility of cigarette use by the teenager. One of psychologists participating in this discussion expressed: 

“The teenagers’ informal groups at school have a great influence on their behaviors. Now, if the conditions of smoking at school are available, saying no to cigarettes will be more difficult for the teenager” (Male, 45 years old, training psychologist).

The unsupervised places of school like teachers’ parking, under maintenance classes, behind playgrounds and places like this where students can commit irregular acts without the fear of being seen by school authorities, can provide extremely effective opportunities to experience cigarettes.

#### 4.3.2. Lack of Protective Programs for Weak Students

Most interviewees believed that absence of protective programs for Weak students (from the physical point of view and regarding emotional skills etc.) are considered as effective causes of cigarette smoking among teenagers. One of the parents expressed his opinion in this regard:

“When the older students annoy my child and put pressure on him, I can’t be with him to defend him, he can’t cope with them” (Female, 50 years old, a teenager’s mother).

#### 4.3.3. Smoking Teachers

Teachers as the students’ role models are considered as another effective factor on students` smoking behavior.

Participant number 29 expressed that “During the break, our teachers would go behind the pantry and smoke” (Male, 13 years old).

Both teenagers and experts expressed that openly smoking inside the school environment, or even out of it by smoking teachers is one of the effective factors on the students’ tendency toward cigarette use. It seems that teachers’ behavior as one of the important role models of the teenage period for students, beyond the instructional programs of anti-tobacco, is effective on students` tendency or lack of tendency toward cigarette use.

### 4.4. Perceived Positive Consequences of Smoking at School

The majority of participants in this study expressed that one of the most important reasons of tendency toward cigarette use among students is the expectation of positive consequences against smoking behavior. The subordinates of this category are three themes explained as follows:

#### 4.4.1. Cigarette Use as a Value Among Students

Students who had experienced cigarettes believed that at their schools, smoking was a superiority factor among students, and was somehow considered as a value among many groups of students. Participant number 23 mentioned that:

“As we wanted the other classmates to think that we are superior to them, we smoked in front of them” (Male, 15 years old).

According to the participants, lack of supervision by headmasters and teachers on forming valuation systems at schools will make smoking and doing high-risk behaviors a value among informal groups of teenagers at school.

#### 4.4.2. Obtaining Permission of Entry to Forbidden Informed Groups at School

According to the participants receiving protection of the group and obtaining permission of entrance to informal groups are mentioned as some other effective reasons in tendency toward cigarette use among teenagers. A teenager who has become a smoker at school mentioned:

One of the cases that lead teenagers to smoking is entry to forbidden groups, increasing the sense of belonging to the group, and receiving supports of the group. In fact, the teenager stabilizes his membership in the forbidden groups formed at school, by cigarette use.

#### 4.4.3. Smoking for Achieving Higher Marks

The intense educational competition, and using different methods to obtain higher marks, is one of the causes that persuade a teenager to use cigarettes. Participant number 18 expressed:

“Some guys smoke to stay awake or to reinforce their memory”(Male, 19 years old).

Another participant said: “It had really affected my studies and my marks improved dramatically.” (Male, 19 years old, number 24)

The comments of experts participating in this research confirmed the students’ opinions about this matter. It seems that intense educational competitions, excessive evaluation of grades, assigning special privileges to elite students, and the discrimination imposed by teachers and school authorities on students in terms of grades are some of the reasons and motivations of students for using abnormal methods like smoking in order to obtain higher scores.

## 5. Discussion

In the current study, some of the participants stated that the presence of high-risk students could increase the possibility of smoking among other students, and cigarettes may persuade them to try other high-risk behaviors. The study of Ziaoddiny et al. also showed that the total prevalence of narcotic substances among boy students is 26.5%, which is statistically high (21). It also showed that use of narcotics among those who smoke is 10 times more than the other students (21). In addition, the study of Habibi et al. showed that 96.6% of smoking students have consumed alcoholic drinks at least once during the last three months (22). Salimi also mentions that 60-70% of students have smoked their first cigarette in the presence of their friends (23). With regard to the fact that a teenager spends long hours at school, and considering the fact that peers greatly influence a teenager (24), it is necessary to pay more attention to screening of high-risk students, and identifying them to prevent more hurt, and protect other students.

Lack of appropriate educational programs and ineffective performance of the available ones require reconsideration of programs, and benefiting from health education experts. The study of Ziaoddiny showed that most of the instructions have been given by the principal and the least training has been done by the health instruction teacher (21), consequently, since non-professional staff have a bigger share in the instruction than the experts, it can cause ineffectiveness of instruction.

Many studies confirm the role of smoking teachers in unwantedly promoting cigarette use among students. Poulsen et al. based on a study in Denmark showed that teachers’ smoking during school hours plays an important role in urging students to smoke (25). Heydari et al. showed that 27.2% of male teachers in Tehran are smokers (26). The study of Charkazi et al. introduced the smoking teacher population as 23.6% (27). With regard to the fact that teachers are among the most effective role models of students (25, 26, 28), the cigarette smoking of the teachers is effective in students’ patterning.

The participants expressed that family partnership is obligatory for success of preventive programs at schools. Other studies confirm this matter. The studies carried out in Iran indicate passivity of Iranian families in the case of educating their children about the harmful effects of tobacco use (29). Zareian’s study showed that the parents’ involvement in educational anti-smoking programs caused a significant decrease in the progressive process of cigarette smoking among teenagers (29), which conforms to the results of the current study.

Many other studies confirm the results of this research about the fact that schools are currently a place for using high risk substances, prohibitions, and anti-smoking rules at school, access to cigarette around the school, prevention of predisposing backgrounds, educational status, and effect of other factors related to schools on the situation of teenage smoking (23, 30-32).

Finally, the researcher suggests that in order to reduce cigarette use in the society, screening programs should be conducted at schools, they should be specifically catered to identifying high-risk schools regarding existence or non-existence of these types of programs, smoking teachers, susceptible students to tobacco use, vulnerable students, high-risk students, and physical predisposing status. These screening programs aid in conducting effective anti-tobacco programs and reducing the possibility of smoking behavior among teenagers, and consequently tobacco use in the society.

The main strong point of this study was to focus on the role of school in initiation of cigarette use among teenagers. The most important limitation of this study was the use of Non-probability Sampling methods and also not to have studied girl groups that limits the generalization of its results.
